# Metronomic chemotherapy with cyclophosphamide and dexamethasone in patients with metastatic carcinoma of the prostate

**DOI:** 10.1038/bjc.2012.78

**Published:** 2012-03-13

**Authors:** P D Dickinson, D N Y Peel, S Sundar

**Affiliations:** 1 Department of Clinical Oncology, Nottingham University Hospitals NHS Trust, Nottingham City Hospital, Hucknall Road, Nottingham NG5 1PB, UK; 2 Department of Clinical Oncology, University Hospitals of Leicester NHS Trust, Leicester Royal Infirmary, Infirmary Square, Leicester LE1 5WW, UK

**Sir**,

We read with interest the article by Khan *et al* (2011), which found that combined metronomic therapy with low-dose cyclophosphamide and methotrexate, combined with celecoxib, did not demonstrate significant activity in patients with advanced cancer. These conclusions are in contrast to our own findings that show clinical and biochemical response to metronomic low-dose cyclophosphamide and dexamethasone in patients with castration refractory metastatic carcinoma of the prostate.

We performed a retrospective audit of 28 patients with metastatic castration refractory carcinoma of the prostate who received cyclophosphamide 50 mg and dexamethasone 2 mg daily, until disease progression. Patient characteristics are provided in Table 1. Almost all patients had been exposed to previous continuous corticosteroid therapy, either as part of standard chemotherapy regimens, or as an independent hormonal treatment.

Response to treatment was determined according to recognised end points in patients with prostate cancer (Scher *et al*, 2008). A total of 13 out of 28 (46%) patients achieved a nadir PSA response below the baseline value (Figure 1). At 12 weeks, 12 out of 28 (43%) patients had a PSA reduction of ⩾25%, 11 out of 28 (39%) had a PSA rise of ⩾25% and 5 out of 28 (18%) had a PSA within 25% of the baseline value. There was no significant association between disease response and the previous use of docetaxel chemotherapy (Student's *t*-test *P*=0.314). The median time to progression (25% increase above PSA nadir value (Scher *et al*, 2008)) was 16 weeks. Four patients had clinical improvement of symptoms.

Treatment was generally well tolerated in a heavily pre-treated group of castration refractory prostate cancer patients, such that toxicities were mild or potentially attributable to the disease process. During treatment, four patients had myelosuppression, defined as anaemia requiring transfusion, and one patient had a suspected myocardial infarction. Two patients died during treatment. The treatment of two patients was interrupted for 8 and 12 weeks because of surgical procedures.

Previous work has also shown treatment response to metronomic cyclophosphamide and dexamethasone in castration refractory prostate cancer; Glode *et al* (2003) published PSA responses according to the PCWG1 guidelines (Bubley *et al*, 1999), showing that 29% of patients had a PSA reduction of ⩾80%, 39% a reduction of 50–79%, 6% a <50% decrease and 26% of patients had a progressive disease (two consecutive PSA rises). Median time to progression was 9 months (36 weeks). As in our series, treatment was well tolerated. In contrast, our patients were more heavily pre-treated and the patients reported here had higher Gleason scores (9 *vs* 8.2).

It is possible that response to metronomic chemotherapy is, in part, influenced by the histology of the primary tumour. The study by Khan *et al* (2011) contained patients with breast, gastrointestinal, renal, melanoma, ovarian, prostate (9 out of 69 patients) and unknown primary tumours. Our work, which shows response to metronomic chemotherapy, alongside the work by Glode *et al* (2003) shows benefit in patients specifically with prostate cancer. We believe that further investigation into the use of metronomic chemotherapy is warranted in prostate cancer, but acknowledge that may not be appropriate in all histological tumour subtypes.

The use of dexamethasone concurrently with cyclophosphamide could have contributed to the response demonstrated in our patients. However, as stated above, virtually all of our patients had been treated previously with corticosteroids, and so we believe the use of cyclophosphamide was responsible for most of the clinical benefit seen.

In conclusion, we believe that metronomic treatment with cyclophosphamide is a well tolerated and useful treatment in heavily pre-treated patients with metastatic castration refractory carcinoma of the prostate, and warrants further investigation.

## Figures and Tables

**Figure 1:**
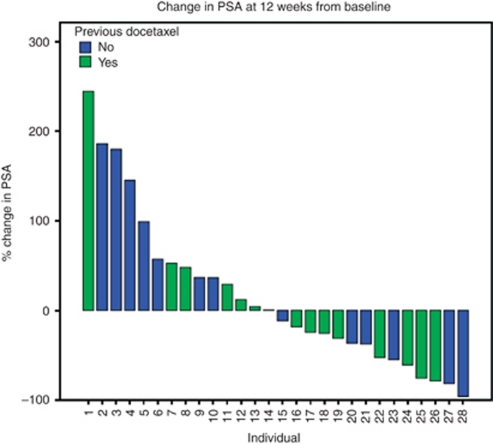
Change in PSA from baseline 12 weeks after the introduction of treatment with cyclophosphamide and dexamethasone.

**Table 1:** Patient characteristics

	**Number of patients**
Mean age (years)	75
*Gleason score*	
• 5	1
• 6	1
• 7	1
• 8	2
• 9	10
• 10	2
• Biopsy not performed	11
	
*Number of previous lines of hormone therapy*	
• 2	2
• 3	15
• 4	8
• 5	3
	
*Number of previous lines of chemotherapy*	
• 0	14
• 1	12
• 2	2
Median baseline PSA at commencement of treatment	123.5 (range 16–3448)

## References

[bib1] Bubley GJ, Carducci M, Dahut W, Dawson N, Daliani D, Eisenberger M, Figg WD, Freidlin B, Halabi S, Hudes G, Hussain M, Kaplan R, Myers C, Oh W, Petrylak DP, Reed E, Roth B, Sartor O, Scher H, Simons J, Sinibaldi V, Small EJ, Smith MR, Trump DL, Vollmer R, Wilding G (1999) Eligibility and response guidelines for phase II clinical trials in androgen-independent prostate cancer: recommendations from the PSA working group. J Clin Oncol 17: 3461–3467 10550143 10.1200/JCO.1999.17.11.3461

[bib2] Glode M, Barqawi A, Crawford E, Kerbel R (2003) Metronomic therapy with cyclophosphamide and dexamethasone for prostate cancer. Cancer 99: 1643–1648 10.1002/cncr.11713 14534880

[bib3] Khan OA, Blann AD, Payne MJ, Middleton MR, Protheroe AS, Talbot DC, Taylor M, Kirichek O, Han C, Patil M, Harris AL (2011) Continuous low-dose cyclophosphamide and methotrexate combined with celecoxib for patients with advanced cancer. Br J Cancer 104: 1822–1827 21587257 10.1038/bjc.2011.154 PMC3111194

[bib4] Scher HI, Halabi S, Tannock I, Morris M, Sternberg CN, Carducci MA, Eisenberger MA, Higano C, Bubley GJ, Dreicer R, Petrylak D, Kantoff P, Basch E, Kelly WK, Figg WD, Small EJ, Beer TM, Wilding G, Martin A, Hussain M, Prostate Cancer Clinical Trials Working Group (2008) Design and end points of clinical trials for patients with progressive prostate cancer and castrate levels of testosterone: recommendations of the prostate cancer clinical trials working group. J Clin Oncol 26: 1148–1159 18309951 10.1200/JCO.2007.12.4487 PMC4010133

